# The Demographic and Clinicopathological Characteristics of Hepatopancreatobiliary Cancers From a Specialized Center in India

**DOI:** 10.7759/cureus.43026

**Published:** 2023-08-06

**Authors:** Puja Sahai, Hanuman P Yadav, Archana Rastogi

**Affiliations:** 1 Department of Radiation Oncology, Institute of Liver and Biliary Sciences, New Delhi, IND; 2 Department of Pathology, Institute of Liver and Biliary Sciences, New Delhi, IND

**Keywords:** liver cancer, hepatopancreatobiliary cancers, hepatocellular carcinoma, gallbladder neoplasms, biliary tract neoplasms

## Abstract

Introduction

Hepatopancreatobiliary (HPB) cancers are a distinct group requiring specialized multidisciplinary care. The present study was an analysis of HPB cancers.

Methods

The institutional data for two years from 2017 to 2018 was retrieved from the hospital-based cancer registry (HBCR) database in Excel format (Microsoft^®^ Corp., Redmond, WA). The demographic details, method of diagnosis, tumor characteristics, and disease extent of the patients with HPB primary sites were retrospectively analyzed.

Results

Out of the 1417 patients with HPB malignancies, 1193 were analyzed. Most of the patients at our center hailed from North India. The distribution of HPB cancers was as follows: hepatocellular carcinoma (HCC) (n=717, 60.1%), gallbladder (GB) (n=230, 19.3%), periampullary carcinoma (n=76, 6.37%), head of the pancreas (HOP) (n=55, 4.61%), extrahepatic bile duct (EBD) (n=53, 4.44%), intrahepatic bile duct (IBD) (n=32, 2.68%), and body of the pancreas (BOP) (n=30, 2.52%). The most common disease presentation of HPB cancers was in the seventh and sixth decades of life. Male predominance was seen in all HPB cancers except in GB with a higher incidence in females. The predominant cause of chronic liver disease (CLD) with HCC was viral-related (53.7%) with hepatitis B virus (HBV) (34.3%) higher than hepatitis C virus (HCV) (19.2%) followed by non-alcoholic fatty liver disease (NAFLD)/non-alcoholic steatohepatitis (NASH) (24.8%) and then alcohol. Adenocarcinoma was the most common morphology in pancreatobiliary malignancies. The disease presentation was locoregional in 63.4% of HCC, 69.7% of periampullary carcinoma, and 50.9% of HOP cases. The patients with EBD and IBD cancers presented commonly with locoregional disease extent in 60.4% and 62.5%, respectively. Perihilar subsite was more commonly detected (71.7%) as compared to the distal one in the patients with EBD cancers. The patients with GB cancers (GBC) presented with distant metastases in 53.5% and locoregional disease in 33%. Distant metastases were present in 76.7% of the patients with BOP cancers. The liver was a common site of distant metastases in GB, periampullary, and pancreatic cancers.

Conclusions

The present study highlights the characteristics and the variations in disease presentation in different primary tumor sites of HPB cancers. In view of the common locoregionally advanced disease presentation of HCC, the patients with CLD need surveillance for the early detection of lesions. As the patients with HPB cancers show advanced disease presentation, effective locoregional and systemic therapies are needed.

## Introduction

Hepatopancreatobiliary (HPB) cancers are a distinct group requiring specialized multidisciplinary care. The incidence of HPB cancers has been increasing in various parts of the world [1,2]. The International Classification of Diseases for Oncology, Third Edition (ICD-O3) code of C22 includes both the liver and intrahepatic bile duct (IBD) as the primary tumor origin, which are often reported together. As per the GLOBOCAN 2020 [1], a total of 905677 new cases were reported to be diagnosed with liver cancer. Liver cancer was the sixth most common cancer and the third leading cause of cancer-related mortality worldwide [1]. The incidence and mortality of liver cancer have been reported to be higher among males as compared with females [3]. Among males, liver cancer was the second leading cause of mortality [3]. There is a geographic variation in the incidence of liver cancer, with the highest rates in countries in Eastern Asia, South-Eastern Asia, and Northern and Western Africa [3].

A total of 115949 cases of gallbladder cancer (GBC) were reported in the GLOBOCAN 2020 with the highest incidence and mortality in Eastern and South-Central Asia [1]. The mortality rate for pancreatic cancer remains high with the highest incidence rates in Europe, North America, and Australia/New Zealand as reported in the Global Cancer Statistics 2020 [3]. The cancers of the extrahepatic bile duct (EBD) and periampullary carcinoma with or without GBC are commonly clubbed together under the broad group of biliary tract neoplasms. The global trends of intrahepatic and extrahepatic cholangiocarcinoma show the highest incidence rates in Asian countries such as South Korea, Thailand, and Japan with increasing rates in Latvia, China, and Colombia [2].

The incidence of cancer in India has been rising as reported from the registries. The highest cancer burden has been reported from the north (2408 disability-adjusted life years {DALYs} adjusted mortality to incidence ratio {AMI}) and northeast regions in India with higher incidence among males [4]. The projected cancer burden in India is expected to increase to 29.8 million DALYs AMI in 2025 [4]. The present study was a retrospective analysis with respect to the demographic and clinicopathological characteristics of each primary tumor site separately in HPB cancers from the hospital-based cancer registry (HBCR) database from India.

## Materials and methods

Our institute has been participating in the multicentric study on the HBCRs under the National Cancer Registry Programme (NCRP) through the Indian Council of Medical Research (ICMR)-National Centre for Disease Informatics and Research (NCDIR), Bengaluru, India. Our center specializes in hepatopancreatobiliary diseases and allied sciences. The Institutional Ethics Committee/Institutional Review Board approval for our institute’s participation in the study was obtained (IEC/2018/58/NA01).

The inclusion criteria were all neoplasms as defined by the ICD-O3 for all sites of cancer. The exclusion criteria were all benign neoplasms, borderline malignancy, carcinoma in situ, and no malignancy. The data was abstracted from the patients and/or medical records and filled in the “patient information forms” provided by the ICMR-NCDIR for all the malignant neoplasms reported/diagnosed/treated at our institute. The data from the completed forms was transmitted and updated regularly through the online HBCR software to the ICMR-NCDIR. The data was monitored with respect to completeness and quality by the cancer registry team at the ICMR-NCDIR. The confidentiality of the participants was maintained, and anonymized data was used for the analysis.

The institutional data for two years from January 1, 2017, to December 31, 2018, was retrieved from the HBCR software in Excel format (Microsoft® Corp., Redmond, WA) and retrospectively analyzed. The patients with HPB malignancies were selected for the present study, and other primary site cancers were excluded. As the present study intended to analyze the data of the patients from India, the patients hailing from the neighboring countries, i.e., Bangladesh, Nepal, Myanmar, Afghanistan, or Bhutan, were excluded from the analysis. The patients with unclassified malignancy diagnosed on imaging, i.e., ultrasonography (USG), contrast-enhanced computed tomography (CECT), contrast-enhanced magnetic resonance imaging (CEMRI), and/or magnetic resonance cholangiopancreatography (MRCP), without further characterization of the lesion were excluded. The patients with a diagnosis of malignant tumor cells on microscopic examination without further morphological classification were excluded too from the analysis. The patients with hepatoblastoma, hemangioendothelioma, hepatic lymphoma, combined hepatocellular cholangiocarcinoma, and biliary tract site unspecified were not included in the present study as the number of cases was few. The demographic details, method of diagnosis, tumor characteristics, and disease extent were analyzed.

The method of diagnosis was noted as microscopic and/or radiological for hepatocellular carcinoma (HCC). The microscopic diagnosis was from cytology, biopsy, and/or the surgical specimen from the tumor. Radiological diagnosis of HCC was based on multiphasic CECT or CEMRI with arterial-phase hyperenhancement and washout on venous or delayed phase as per the American Association for the Study of Liver Diseases (AASLD) guidelines [5,6]. Chronic liver disease (CLD) was diagnosed from the patient’s history, physical examination, laboratory tests, and imaging [7,8]. Non-cirrhotic liver was diagnosed from the patient’s history, physical examination, laboratory tests, and imaging with or without pathological confirmation from the surgical specimen. The microscopic confirmation of HCC was obtained in the patients with non-cirrhotic liver. Some of the patients with radiological confirmation of HCC had undergone cytology/cell block or biopsy for immunohistochemistry markers. The other HPB cancers were diagnosed microscopically from the primary tumor and/or metastatic site.

The disease extent at diagnosis was noted from the clinical examination and imaging and classified into localized, locoregional, and distant metastases. Locoregional extent was defined as local spread with direct extension into adjacent organs or structures and/or regional lymph node involvement. The regional lymph nodes and distant metastatic sites for the respective HPB cancers were defined as per the American Joint Committee on Cancer (AJCC) staging (seventh or eighth edition) [9,10] for the patients diagnosed in 2017 and 2018, respectively. The descriptive data was expressed as frequencies and proportions.

## Results

**Figure 1:**
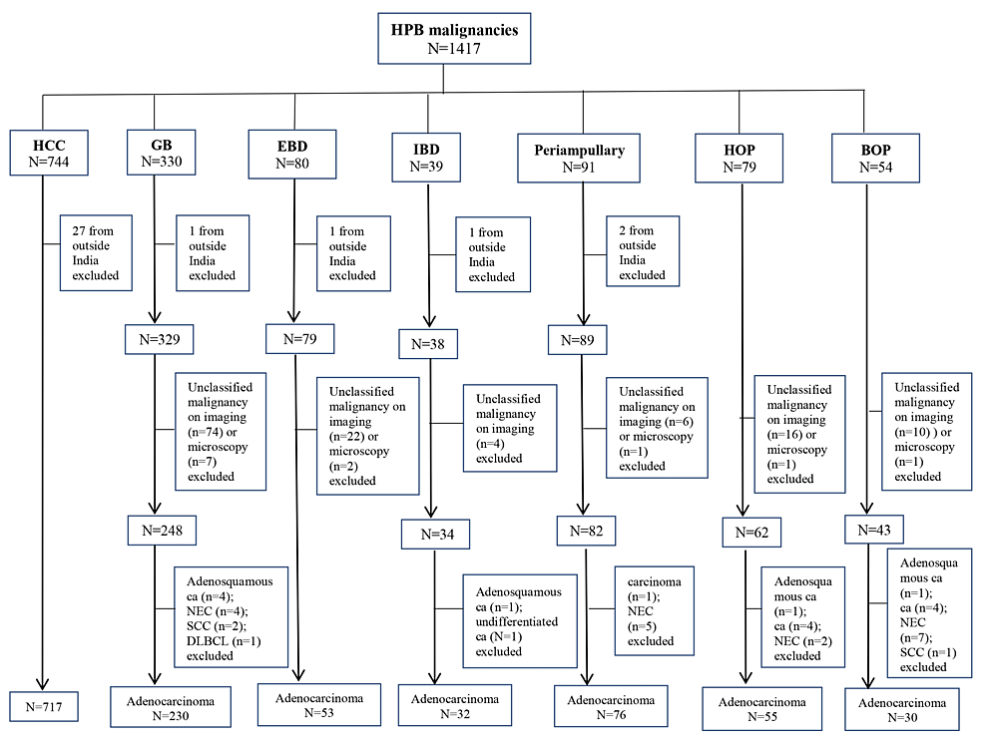
Flowchart depicting the distribution of hepatopancreatobiliary (HPB) malignancies HCC, hepatocellular carcinoma; GB, gallbladder; EBD, extrahepatic bile duct; IBD, intrahepatic bile duct; HOP, head of the pancreas; BOP, body of the pancreas; ca, carcinoma; NEC, neuroendocrine carcinoma; SCC, squamous cell carcinoma; DLBCL, diffuse large B-cell lymphoma

**Figure 2:**
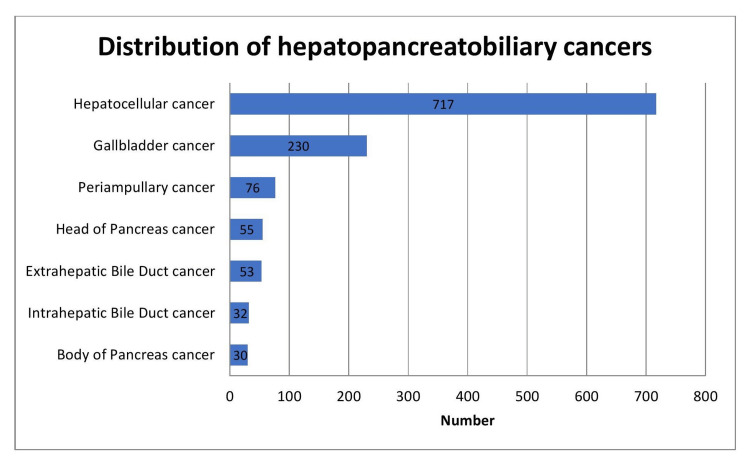
Chart illustrating the distribution of hepatopancreatobiliary cancers

A total of 1417 patients were diagnosed with HPB malignancies. Out of the 1417 patients, 1193 were analyzed. The flowchart depicting the distribution of HPB malignancies is demonstrated in Figure 1. The distribution of HPB cancers is illustrated in Figure 2.

Hepatocellular carcinoma

HCC constituted the major primary site of HPB cancers (717 out of 1193, 60.1%). Out of the 717 patients from India, the majority (n=191, 26.6%) hailed from North India in Uttar Pradesh (n=168) and the National Capital Territory of Delhi (n=167). The details of the patients are mentioned in Table 1. At disease presentation, the majority of the patients belonged to the seventh followed by the sixth decade of life with the age ranging from 14 years to 85 years. CECT was the most common modality for the diagnosis and staging on imaging (423 out of 543). Ten patients were diagnosed on USG with or without contrast and/or hepatic vein Doppler with raised tumor marker as further CECT or CEMRI could not be performed due to renal derangement and advanced cirrhosis. Of the 543 patients, 60 had undergone 18-fluorine-fluorodeoxyglucose positron emission tomography-contrast-enhanced computed tomography (18F-FDG PET-CECT) along with the triphasic CECT or CEMRI at diagnosis. PET-CECT was performed for discerning the disease extent and avidity and workup for distant metastasis. Of the patients who had pathological diagnosis (n=174), 130 had undergone CECT and 25 undergone CEMRI for staging. Of the 130 patients with CECT, 32 had undergone PET-CECT additionally for staging. In the remaining 19 patients, information about the imaging modality used was not available from the medical records. Of the 483 patients with locoregional disease, 73 had regional lymph nodes. Among patients with distant metastasis at presentation, the most common site was the lung (n=38) followed by the bone (n=12). The other secondary sites were peritoneum, adrenal gland, and distant lymph nodes with or without lung or bone lesions.

**Table 1:** Characteristics of the patients with hepatocellular carcinoma HCC, hepatocellular carcinoma; CECT, contrast-enhanced computed tomography; CEMRI, contrast-enhanced magnetic resonance imaging

Characteristic	N (%)
Patients diagnosed with hepatocellular carcinoma	717
Patients with microscopic diagnosis of HCC	174 (24.3)
Histology of primary tumor	73
Cytology of primary tumor	96
Histology of metastatic lesion	2
Cytology of metastatic lesion	3
Patients with the diagnosis of HCC on imaging	543 (75.7)
CECT	423
CEMRI	72
CECT+CEMRI	34
Ultrasonography	10
Information not available	4
Age (years)	
10-19	2 (0.28)
20-29	14 (1.95)
30-39	22 (3.07)
40-49	74 (10.3)
50-59	214 (29.9)
60-69	273 (38.1)
70-79	104 (14.5)
>80	14 (1.95)
Gender	
Male	621 (86.6)
Female	96 (13.4)
Disease extent at presentation	
Localized	139 (19.4)
Locoregional	483 (63.4)
Distant metastasis	74 (10.3)
Information not available	21 (2.93)

Of the 717 patients, 642 had CLD, while 60 had non-cirrhotic liver. In 15 out of the 717 patients, inadequate information was available on the status of the liver as the patients were diagnosed and/or partially treated elsewhere before registration at our institute. Of the 642 patients, the etiology of CLD was documented in 557, while the remaining 85 had not received complete workup to define the cause of CLD. CLD was viral-related in 299 (53.7%) out of the 557 patients with HCC. Hepatitis B virus (HBV) was the leading cause of CLD in 191 (34.3%) of the 557 patients, while hepatitis C virus (HCV) positivity was documented in 107 (19.2%) patients. Both HBV and HCV were the causes of CLD in one patient. Eleven out of the 299 patients with viral CLD had associated non-alcoholic steatohepatitis (NASH) (n=6) or alcoholic liver disease (ALD) (n=5). Non-alcoholic fatty liver disease (NAFLD)/NASH was the cause of CLD in 138 (24.8%) patients with HCC. In the remaining patients, the cause of CLD was as follows: ALD (n=97, 17.4%) and cryptogenic liver disease (n=20). Three out of the 557 patients had autoimmune hepatitis with two of them having primary biliary cholangitis as the cause of CLD.

Gallbladder (GB)

Among the patients with gallbladder adenocarcinoma, 191 (83%) out of the 230 hailed from North India from the following regions: National Capital Territory of Delhi (n=73), Uttar Pradesh (n=59), and Uttarakhand (n=59). Of the 230 patients, 41 (17.8%) had presented after laparoscopic cholecystectomy with incidental detection of malignancy of the gallbladder. Laparoscopic cholecystectomy was performed for benign gallbladder disease, i.e., gallstone disease or cholecystitis. The details of the 230 patients are mentioned in Table 2. At disease presentation, the majority of the patients belonged to the sixth (33.5%) followed by the seventh (30.9%) decade of life with the age ranging from 25 years to 87 years. Of the 76 patients with locoregional disease, 56 had regional lymph nodes. More than half (53.5%) of the patients presented with distant metastasis. The liver alone or in combination with other sites was the most common site of distant metastasis at presentation (89 out of 123, 72.4%).

**Table 2:** Characteristics of the patients with gallbladder carcinoma

Characteristic	N (%)
Patients with adenocarcinoma as microscopic morphology	230
Method and site of microscopic diagnosis	
Histology of primary tumor	74 (32.2)
Cytology of primary tumor	80 (34.8)
Histology of metastatic lesion	16 (6.96)
Cytology of metastatic lesion	60 (26.1)
Age (years)	
20-29	5 (2.17)
30-39	18 (7.83)
40-49	39 (17)
50-59	77 (33.5)
60-69	71 (30.9)
70-79	19 (8.26)
>80	1 (0.43)
Gender	
Male	90 (39.1)
Female	140 (60.9)
Disease extent at presentation	
Localized	17 (7.39)
Locoregional	76 (33)
Distant metastasis	123 (53.5)
Information not available	14 (6.19)

Extrahepatic bile duct

Nearly half of the patients hailed from the state of Uttar Pradesh (23 out of 53) with the remaining from the National Capital Territory of Delhi, Haryana, Madhya Pradesh, Bihar, Jharkhand, Manipur, and other states. The characteristics of 53 patients diagnosed with adenocarcinoma microscopically are listed in Table 3. The age of the patients with extrahepatic cholangiocarcinoma ranged from 25 years to 80 years with the majority belonging to the fifth decade (40-49 years) of life at disease presentation. Perihilar subsite was more commonly detected as compared to the distal one. Of the 32 patients with locoregional disease, 16 had regional lymph nodes.

**Table 3:** Characteristics of the patients with extrahepatic bile duct carcinoma

Characteristic	N (%)
Patients with adenocarcinoma as microscopic morphology	53
Method and site of microscopic diagnosis	
Histology of primary tumor	20 (37.7)
Cytology of primary tumor	24 (45.3)
Histology of metastatic lesion	2 (3.77)
Cytology of metastatic lesion	7 (13.2)
Age (years)	
20-29	1 (1.9)
30-39	2 (3.77)
40-49	20 (37.7)
50-59	12 (22.6)
60-69	10 (18.9)
70-79	6 (11.3)
>80	2 (3.77)
Gender	
Male	30 (56.6)
Female	23 (43.4)
Disease extent at presentation	
Localized	4 (7.55)
Locoregional	32 (60.4)
Distant metastasis	15 (28.3)
Information not available	2 (3.77)
Subsite	
Perihilar	38 (71.7)
Distal	6 (11.3)
Unspecified	9 (17)

Intrahepatic bile duct

Of the 32 patients with intrahepatic cholangiocarcinoma, most hailed from the National Capital Territory of Delhi (n=10) and Uttar Pradesh (n=6) and the remaining from Madhya Pradesh, Jammu and Kashmir, Rajasthan, Arunachal Pradesh, and other states from India. The details of the 32 patients are listed in Table 4. The patients’ age ranged from 31 to 78 years with the majority presenting in the sixth decade (50-59 years age group) of life. Of the 20 patients with locoregional disease, seven had regional lymph nodes.

**Table 4:** Characteristics of the patients with intrahepatic bile duct carcinoma

Characteristic	N (%)
Patients with adenocarcinoma as microscopic morphology	32
Method and site of microscopic diagnosis	
Histology of primary tumor	19 (59.4)
Cytology of primary tumor	6 (18.8)
Histology of metastatic lesion	5 (15.6)
Cytology of metastatic lesion	2 (6.25)
Age (years)	
20-29	0
30-39	1 (3.13)
40-49	5 (15.6)
50-59	13 (40.6)
60-69	9 (28.1)
70-79	4 (12.5)
>80	0
Gender	
Male	21 (65.6)
Female	11 (34.4)
Disease extent at presentation	
Localized	1 (3.13)
Locoregional	20 (62.5)
Distant metastasis	11 (34.4)

Periampullary

Of the 76 patients, most hailed from the National Capital Territory of Delhi (n=24), Uttar Pradesh (n=22), and Bihar (n=15) with the remaining from other states in India. The details of the 76 patients with microscopic confirmation of periampullary carcinoma are listed in Table 5. The patients presented at ages ranging from 25 to 80 years with most of the patients presenting at the seventh and sixth decades of life. Of the 53 patients with locoregional disease, 23 had regional lymph nodes. The liver was the most common secondary site (in 11 out of 12) in the patients presenting with distant metastasis.

**Table 5:** Characteristics of the patients with periampullary carcinoma CBD: common bile duct

Characteristic	N (%)
Patients with adenocarcinoma as microscopic morphology	76
Method and site of microscopic diagnosis	
Histology of primary tumor	63 (82.9)
Cytology of primary tumor	7 (9.21)
Cytology of metastatic lesion	6 (7.9)
Age (years)	
30-39	9 (11.8)
40-49	12 (15.8)
50-59	22 (29)
60-69	25 (33)
70-79	7 (9.21)
>80	1 (1.32)
Gender	
Male	60 (79)
Female	16 (21.1)
Disease extent at presentation	
Localized	2 (2.63)
Locoregional	53 (69.7)
Distant metastasis	12 (15.8)
Information not available	9 (11.8)
Subsite	
Ampulla of Vater	24 (31.6)
Distal CBD	5 (6.58)
Ampulla+distal CBD	4 (5.26)
Periampullary (subsite unspecified)	43 (56.6)

Head of the pancreas (HOP)

Of the 55 patients, most hailed from the National Capital Territory of Delhi (n=19) and Uttar Pradesh (n=15) with the remaining from other states in India. The details of the 55 patients with the microscopic confirmation of adenocarcinoma of the head of the pancreas (HOP) are listed in Table 6. The patients presented at the ages ranging from 32 to 74 years with most of the patients presenting at the seventh and sixth decades of life. Of the 28 patients with locoregional disease, 19 had regional lymph nodes. The liver was the most common secondary site (in 18 out of 19) in the patients presenting with distant metastasis.

**Table 6:** Characteristics of the patients with head of the pancreas carcinoma

Characteristic	N (%)
Patients with adenocarcinoma as microscopic morphology	55
Method and site of microscopic diagnosis	
Histology of primary tumor	21 (38.2)
Cytology of primary tumor	22 (40)
Histology of metastatic lesion	2 (3.64)
Cytology of metastatic lesion	10 (18.2)
Age (years)	
30-39	4 (7.27)
40-49	10 (18.2)
50-59	19 (34.6)
60-69	18 (32.7)
70-79	4 (7.27)
>80	0
Gender	
Male	43 (78.2)
Female	12 (21.8)
Disease extent at presentation	
Localized	5 (9)
Locoregional	28 (50.9)
Distant metastasis	19 (34.6)
Information not available	3 (5.46)

Body of the pancreas (BOP)

Of the 30 patients, most hailed from the National Capital Territory of Delhi (n=9) and Uttar Pradesh (n=7) with the remaining from other states in India. The details of the 30 patients with the microscopic confirmation of adenocarcinoma of the body of the pancreas (BOP) are listed in Table 7. The patients presented at ages ranging from 24 to 89 years with most of the patients presenting at the seventh and sixth decades of life. Of the five patients with locoregional disease, two had regional lymph nodes. The liver was the most common secondary site (in 21 out of 23) in the patients presenting with distant metastasis.

**Table 7:** Characteristics of the patients with body of the pancreas carcinoma

Characteristic	N (%)
Patients with adenocarcinoma as microscopic morphology	30
Method and site of microscopic diagnosis	
Histology of primary tumor	2 (6.67)
Cytology of primary tumor	13 (43.3)
Histology of metastatic lesion	3 (10)
Cytology of metastatic lesion	12 (40)
Age (years)	
20-29	1 (3.33)
30-39	0
40-49	4 (13.3)
50-59	8 (26.7)
60-69	10 (33.3)
70-79	5 (16.7)
>80	2 (6.67)
Gender	
Male	23 (76.7)
Female	7 (23.3)
Disease extent at presentation	
Localized	1 (3.33)
Locoregional	5 (16.7)
Distant metastasis	23 (76.7)
Information not available	1 (3.33)

Summary findings on HPB cancers

The major group of the patients with HPB cancers consulting at our center hailed from North India. The most common disease presentation was observed in the seventh and sixth decades of life for the HPB cancers. Male predominance was observed in all HPB cancers except for gallbladder cancers with a higher incidence in females. HCC was the most common primary site (60.1%) among the HPB group of cancers. The predominant cause of CLD with HCC was viral-related (53.7%) with HBV (34.3%) higher than HCV (19.2%) followed by NASH (24.8%) and then alcohol. The gallbladder was the most common site among biliary tract cancers (BTCs). Adenocarcinoma was the most common morphology in pancreatobiliary malignancies. The disease presentation was locoregional in 63.4% of HCC, 69.7% of periampullary, and 50.9% of head of the pancreas cases. The patients with extrahepatic and intrahepatic bile duct cancers presented commonly with locoregional disease extent. Perihilar subsite was more commonly detected as compared to the distal one in EBD cancers. More than half (53.5%) of the patients with gallbladder cancers presented with distant metastases, while 33% presented with locoregional disease. Distant metastases were noted in 76.7% of the patients with body of the pancreas cancers. The liver was a common site of distant metastases in gallbladder, periampullary, and pancreatic cancers.

## Discussion

The present study described the characteristics of different primary sites of HPB cancers. There has been a trend of changing epidemiology of HCC from viral to nonviral etiologies in recent years across different geographic regions at varying rates. The predominant risk factor for HCC has been HBV in Asia and NAFLD/NASH and alcohol in Europe and North America [11]. The most common etiology for cirrhosis has been identified as HBV in patients with HCC from India [12,13]. However, there is a rising trend in NASH being reported as the etiology of cirrhosis with HCC in some parts of the world. In an institutional experience from Italy [14], a significantly higher proportion of NASH-related HCC was observed in recent years as compared to the past decade. In the present study, the predominant cause of cirrhosis with HCC was viral-related with HBV higher than HCV followed by NASH and then alcohol.

Most of the patients with HCC presented with locoregional advanced disease in the present study. HCC was mainly diagnosed on imaging with multiphasic CECT or CEMRI in patients with CLD. The end stage of CLD is cirrhosis, which occurs after years or decades of the progression of liver damage [7]. It has been established with research that liver fibrosis is a dynamic process and early cirrhosis may be reversible [15]. The goals of the treatment of CLD are to prevent cirrhosis, decompensation, and death [15]. By treating the underlying cause in patients with CLD, improvement may be seen in fibrosis [15]. Most of the HCCs occur in cirrhotic liver. Thus, by preventing cirrhosis, HCC may be prevented too. The current recommendation for screening for HCC is an ultrasound every six months in patients with cirrhosis [6]. In the present study, extrahepatic metastases were seen in 10.3% of the patients with HCC with the figures reported as 13%-19% of the patients from other studies published in India [12,13].

A total of 16399 patients were reported with biliary tract malignancies including GBC from India in the recent report of HBCR from ICMR-NCDIR [16]. There has been an increasing trend in the newly diagnosed GBC cases in urban Delhi as reported from the population-based cancer registry from 1988 to 2012 [17]. Metastatic disease presentation is most commonly observed in patients with GBC [18,19] as was seen in the present study. Epidemiological studies show age, female gender, obesity, congenital biliary tract anomalies, and genetic predisposition representing as risk factors for the development of GBC [20,21]. Environmental triggers leading to chronic inflammation from cholelithiasis or biliary tract parasitic and bacterial infections play a role in developing GBC [20,21]. In the present study, 17.8% of GBC cases were diagnosed incidentally after laparoscopic cholecystectomy. It is important that all gallbladder specimens be opened and suspicious areas sent for frozen section to prevent missing malignancy [22]. Additionally, all gallbladder specimens should be examined histopathologically after cholecystectomy performed for benign gallbladder disease. The most common morphology has been reported as adenocarcinoma in biliary tract and pancreatic malignancies [16,23] similar to the present study.

A population-based cohort study on BTCs from the Swedish Cancer Register reported rising incidence of intrahepatic and extrahepatic cholangiocarcinoma especially in younger adults and a decrease in GBC [24]. An increased risk of HPB cancers has been demonstrated during a median follow-up of 10 years in the patients with inflammatory bowel disease (IBD) with concomitant primary sclerosing cholangitis (PSC) [25]. Estimates from the International Agency for Research on Cancer (IARC) GLOBOCAN database have revealed a decline in the incidence of GBC and a higher proportion of unspecified BTCs in the American continent [26].

Most studies pool the subtypes of BTCs together; however, variations in terms of epidemiology, clinical presentation, response to treatment, prognosis, and molecular alterations are being recognized in the subtypes in the scientific literature [27]. Therefore, the subtypes of BTCs need to be evaluated separately. Multicentric studies may be planned for the separate primary tumor sites in HPB cancers. The present study represents a specific distribution of HPB cancers and provides data for planning management protocols and studies.

The limitations of the study are that the treatment and outcome details of the patients were not included. A further study may be planned to evaluate survival outcomes with the different treatment modalities for the respective primary sites of HPB cancers.

## Conclusions

The present study highlights the characteristics and variations in disease presentation in different primary tumor sites of HPB cancers. In view of the common locoregionally advanced disease presentation of HCC, patients with CLD need surveillance for the early detection of lesions so as to be offered curative treatments. Given the high burden of liver involvement as primary tumor with a background of CLD or metastatic disease in HPB cancers, effective liver-directed cancer and supportive care interventions are needed. As patients with HPB cancers show advanced disease presentation, effective locoregional and systemic therapies are needed.

## References

[1] (2023). All cancers. https://gco.iarc.fr/today/data/factsheets/cancers/39-All-cancers-fact-sheet.pdf.

[2] Florio AA, Ferlay J, Znaor A (2020). Global trends in intrahepatic and extrahepatic cholangiocarcinoma incidence from 1993 to 2012. Cancer.

[3] Sung H, Ferlay J, Siegel RL, Laversanne M, Soerjomataram I, Jemal A, Bray F (2021). Global cancer statistics 2020: GLOBOCAN estimates of incidence and mortality worldwide for 36 cancers in 185 countries. CA Cancer J Clin.

[4] Kulothungan V, Sathishkumar K, Leburu S (2022). Burden of cancers in India - estimates of cancer crude incidence, YLLs, YLDs and DALYs for 2021 and 2025 based on National Cancer Registry Program. BMC Cancer.

[5] Bruix J, Sherman M (2011). Management of hepatocellular carcinoma: an update. Hepatology.

[6] Marrero JA, Kulik LM, Sirlin CB (2018). Diagnosis, staging, and management of hepatocellular carcinoma: 2018 practice guidance by the American Association for the Study of Liver Diseases. Hepatology.

[7] Wiegand J, Berg T (2013). The etiology, diagnosis and prevention of liver cirrhosis: part 1 of a series on liver cirrhosis. Dtsch Arztebl Int.

[8] Starr SP, Raines D (2011). Cirrhosis: diagnosis, management, and prevention. Am Fam Physician.

[9] (2010). AJCC cancer staging manual, seventh edition.

[10] (2017). AJCC cancer staging manual, eighth edition. Brookland RK, Washington MK, Gershenwald JE, et al.: AJCC Cancer Staging Manual. 8th ed.

[11] Vogel A, Meyer T, Sapisochin G, Salem R, Saborowski A (2022). Hepatocellular carcinoma. Lancet.

[12] Shukla A, Patkar S, Sundaram S (2022). Clinical profile, patterns of care & adherence to guidelines in patients with hepatocellular carcinoma: prospective multi-center study. J Clin Exp Hepatol.

[13] Kumar R, Saraswat MK, Sharma BC, Sakhuja P, Sarin SK (2008). Characteristics of hepatocellular carcinoma in India: a retrospective analysis of 191 cases. QJM.

[14] Giannitrapani L, Zerbo M, Amodeo S (2020). The changing epidemiology of hepatocellular carcinoma : experience of a single center. Biomed Res Int.

[15] Smith A, Baumgartner K, Bositis C (2019). Cirrhosis: diagnosis and management. Am Fam Physician.

[16] (2023). Clinicopathological profile of cancers in India: a report of the hospital based cancer registries, 2021. https://ncdirindia.org/All_Reports/HBCR_2021/Default.aspx.

[17] Malhotra RK, Manoharan N, Shukla NK, Rath GK (2017). Gallbladder cancer incidence in Delhi urban: a 25-year trend analysis. Indian J Cancer.

[18] Dubey AP, Rawat K, Pathi N, Viswanath S, Rathore A, Kapoor R, Pathak A (2018). Carcinoma of gall bladder: demographic and clinicopathological profile in Indian patients. Oncol J India.

[19] Singh S, Bhatnagar S, Lohia N (2022). Epidemiological and geographical profile of gall bladder cancer patients from a hospital-based registry of northern gangetic plains. Asian Pac J Environ Cancer.

[20] Hundal R, Shaffer EA (2014). Gallbladder cancer: epidemiology and outcome. Clin Epidemiol.

[21] Nemunaitis JM, Brown-Glabeman U, Soares H (2018). Gallbladder cancer: review of a rare orphan gastrointestinal cancer with a focus on populations of New Mexico. BMC Cancer.

[22] Rathanaswamy S, Misra S, Kumar V, Chintamani Chintamani, Pogal J, Agarwal A, Gupta S (2012). Incidentally detected gallbladder cancer- the controversies and algorithmic approach to management. Indian J Surg.

[23] Shakuntala ST, Krishnan SK, Das P (2022). Descriptive epidemiology of gastrointestinal cancers: results from National Cancer Registry Programme, India. Asian Pac J Cancer Prev.

[24] Rahman R, Ludvigsson JF, von Seth E, Lagergren J, Bergquist A, Radkiewicz C (2022). Age trends in biliary tract cancer incidence by anatomical subtype: a Swedish cohort study. Eur J Cancer.

[25] Yu J, Refsum E, Helsingen LM (2022). Risk of hepato-pancreato-biliary cancer is increased by primary sclerosing cholangitis in patients with inflammatory bowel disease: a population-based cohort study. United European Gastroenterol J.

[26] Miranda-Filho A, Piñeros M, Ferreccio C, Adsay V, Soerjomataram I, Bray F, Koshiol J (2020). Gallbladder and extrahepatic bile duct cancers in the Americas: incidence and mortality patterns and trends. Int J Cancer.

[27] Valle JW, Kelley RK, Nervi B, Oh DY, Zhu AX (2021). Biliary tract cancer. Lancet.

